# Co-infection of hepatitis E virus, *Clonorchis sinensis*, and *Escherichia coli*: A case report

**DOI:** 10.3389/fcimb.2023.1078870

**Published:** 2023-03-07

**Authors:** Lu Zhang, Xiaohao Wang, Jing Zhang, Zhongrong Wang, Dachuan Cai

**Affiliations:** ^1^ Department of Infectious Diseases, Key Laboratory of Molecular Biology for Infectious Diseases (Ministry of Education), Institute for Viral Hepatitis, The Second Affiliated Hospital, Chongqing Medical University, Chongqing, China; ^2^ Department of pathogen biology, Basic College of Medicine, Chongqing Medical University, Chongqing, China; ^3^ Department of Gastroenterology, Tongliang District People’s Hospital, Chongqing, China

**Keywords:** hepatitis E virus, HEV, *Clonorchis sinensis*, co-infection, *Escherichia coli*

## Abstract

Hepatitis E virus (HEV) is a common cause of acute hepatitis that threatens human health worldwide. With the popularization of detection technology, the reports of hepatitis E have gradually increased. Here, we present a rare case of co-infection with hepatitis E viruses, *Clonorchis sinensis* and *Escherichia coli*. A 52-year-old man was hospitalized because of fatigue, jaundice, and nausea for more than 2 weeks. Laboratory tests showed elevated bilirubin, aminotransferase (ALT), and aspartate aminotransferase (AST); HEV-IgM was positive, and HEV-RNA could be detected. Moreover, parasites were found in the biliary drainage and the biliary culture, which suggested *Escherichia coli*. The patient was effectively treated with praziquantel, imipenem, and hepatoprotective drugs and his clinical symptoms were relieved after 2 months; total bilirubin decreased to 85.1 μmol/L, ALT decreased to 92.4 U/L, and AST decreased to 102 U/L.

## Introduction

Hepatitis E virus is a positive-sense single-stranded RNA virus that was discovered in the early 1980s to cause hepatitis epidemics. It is one of the five well-known common hepatitis viruses including hepatitis B and hepatitis C and is classified into eight distinct genotypes (GTs) (Zhou et al., 2019). GT1-4 and GT7 are reported to be threatening to humans. According to the WHO, HEV causes 20 million new infections annually, with more than 3 million cases of acute hepatitis and more than 55,000 deaths (World Health Organization, 2016). Africa and Asia have a higher prevalence of HEV (Kmush et al., 2013; Yazbek et al., 2016),with seroprevalence rates in Africa, Asia, Europe, and the North and South America of 21.76%, 15.8%, 9.31%, 8.05%, and 7.28%, respectively (Li et al., 2020).

HEV is usually transmitted through infected food and water or mother to child, but iatrogenic transmission is also possible. Although human-to-human transmission is rare, infected individuals are contagious during viral fecal shedding. HEV infection usually presents with jaundice, fatigue, anorexia, nausea, vomiting, abdominal pain, fever, and hepatomegaly (Harun-Or-Rashid et al., 2013), and other features include diarrhea, arthralgia, pruritus, and urticarial rash (Jameel et al., 1992; Centers for Disease Control and Prevention (CDC), 1993).

*Clonorchis sinensis*, also known as Chinese liver fluke, is prevalent in the Far East. It is a zoonotic parasitic disease estimated to infect more than 23 million people worldwide, including 15 million in China (Andrews et al., 2008; Lai et al., 2016). The prevalence of *Clonorchis sinensis* varies widely in different endemic regions; for example, in different provinces of China, it ranges from less than 1% to 57% (Mahanty et al., 2011). Animals or humans can be infected by ingesting raw, undercooked, salted, cured, or smoked fresh-water fish.

Some patients, especially those with heavy adult worm loads, may develop chronic infection with symptoms including fatigue, abdominal discomfort, anorexia, weight loss, indigestion, and diarrhea (Pungpak et al., 1994). The gallbladder becomes dysfunctional and enlarged, and dead parasites or eggs may become the core of the stone formation. In severe cases, obstructive jaundice, pancreatitis, recurrent cholangitis, and a bacterial liver abscess may occur. More serious complications include cholangitis, cholangiohepatitis, and cholangiocarcinoma (Keiser and Utzinger, 2005; Chang et al., 2021). Praziquantel is the first-line anti-parasitic drug, and albendazole can be used as a second-line anthelmintic therapy.

## Materials and methods

HEV was determined in serum samples using the Anti-HEV ELISA (IgM) Diagnostic Kit (Shanghai Kehua Bio-Engineering Co., Ltd, China) according to the manufacturer’s instructions. The kit was used for qualitative detection of anti-HEV-IgM in serum or plasma with a negative threshold value of 0.000–1.000S/co. Serum HEV RNA was quantified using the Hepatitis E Virus RNA Detection Kit (Fluorescence PCR) (Acon Biotech, Hangzhou Co., Ltd, China).

The sample of gallbladder secretions was inoculated on Columbia blood AGAR plate and cultured in a 35°C incubator (THERMO) for 24 h. The strain was identified and tested for bacterial susceptibility using the VitEK2-Compact automatic microbial analysis system. The results were determined according to the 2020 CLSI guideline. The reagent used is special for the instrument.

The diagnosis of HEV infection was confirmed by HEV IgM positivity. Adult parasites in the biliary drainage and bile cultures suggesting *Escherichia coli* were diagnosed as *Clonorchis sinensis* infection and *E. coli* infection, respectively.

## Case presentation

A 52-year-old man was hospitalized because of fatigue and jaundice accompanied by upper abdominal pain, nausea, retching, and itchy skin for more than 2 weeks. He lives in a town with abundant water in southwest China, works as a freelancer who likes fishing, and has a history of drinking untreated water. He rarely drinks alcohol and has no history of drug use or viral hepatitis. He had not travelled in the past year or eaten raw fish or pork. The physical examination revealed yellow staining of the skin and sclera and tenderness in the upper abdomen.

**Figure 1:**
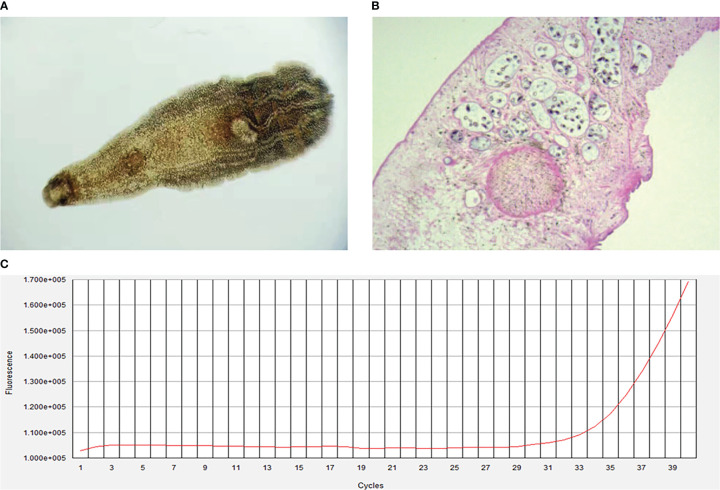
Clonorchis sinensis by microscope **(A)**, original magnification, ×40) and pathology **(B)**, original magnification, ×100). **(C)**, Fluorescence amplification curves of HEV RNA.

The initial blood tests indicated elevated bilirubin and transaminitis (alanine aminotransferase 2173 U/L, aspartate aminotransferase 2592 U/L, γ-glutamyltransferase 99 U/L, alkaline phosphatase 171 U/L, and total bilirubin 313.6 (μmol/L) (**Table 1**). Hepatitis A, B, C, and D antibody tests were negative, while hepatitis E antibody IgM was 8.086S/co, which might indicate HEV infection. HEV infection was confirmed by PCR (**Figure 1C**). The TORCH (Toxoplasma, Rubella Virus, Cytomegalovirus, Herpes Virus), Epstein–Barr virus, and autoimmune liver antibody spectrum tests were negative, and his thyroid function test was normal. The CT scan demonstrated cholecystolithiasis. Endoscopic retrograde cholangiopancreatography and continuous endoscopic nasobiliary drainage were arranged to clarify the cause of jaundice. Unexpectedly, parasites were found in the biliary drainage and identified as *Clonorchis sinensis* by microscope (**Figure 1A**) and pathology (**Figure 1B**). The patient was initially diagnosed with *Clonorchis sinensis* and HEV co-infection, so he was prescribed 0.4g albendazole orally once a day for 2 weeks, and hepatoprotective drugs were also taken.

**Table 1:** Laboratory findings of the patient throughout different stages.

	0week	4weeks	8weeks	12weeks
Hemoglobin g/L	125	90	79	81
WBC count /L	10.68×10^9^	9.23×10^9^	11.91×10^9^	7.5×10^9^
Platelet count u/l	447	246	584	344
ALT U/L	2173	60	104	92.4
AST U/L	2592	82	196	102
ALP U/L	171	102	97	114.3
GGT U/L	99	62	80	126.4
Bilirubin total mg/dl	313.6	413.1	299.5	85.1
BUN mg/dl	8.76	4.39	4.06	–
Creatinine mg/dl	64.3	56.8	45.2	–
PTA %	84.5	73	68	77.7
INR	1.1	1.22	1.27	1.14
Lac	3.02	2.92	3.86	–

- represents missing data.

However, his bilirubin progressively increased despite reduced transaminases (**Table 1**). The abdominal ultrasound depicted gallbladder cholestasis, gallbladder stones, and gallbladder wall thickening, and MRCP was taken as well to confirm the diagnosis of gallstones, cholecystitis, and cholestasis in the follow-up. Given that the parasites were repeatedly found in the nasobiliary drainage, he was administered 25 mg/kg praziquantel thrice daily for a total of 3 days for deworming. He developed fever and abdominal pain and a physical examination demonstrated tenderness and rebound tenderness in the right upper abdomen, so he was considered to have acute exacerbation of cholecystitis. The bile culture demonstrated *E.coli* which is sensitive to imipenem and his symptoms improved and bilirubin decreased after imipenem treatment. Twelve weeks after presentation, the patient’s symptoms had stabilized, the total bilirubin decreased to 85.1 μmol/L, ALT decreased to 92.4 U/L, AST decreased to 102 U/L, and GGT decreased to 126.4 U/L (**Table 1**).

## Discussion

This paper reports a case of triple infection with the hepatitis E virus, *Clonorchis sinensis* and *E. coli*. In this case, the patient had not visited an epidemic area or had any contact with infected animals, but had a history of drinking untreated water. He was diagnosed with an acute HEV infection due to his epidemiological history and typical manifestations such as jaundice, fatigue, abdominal pain, fever, HEV IgM, and HEV RNA positivity.

HEV infection is one of the common causes of acute hepatitis and is a mild self-limiting disease in immunocompetent patients, but some cases may develop into a chronic infection, even liver cirrhosis and liver failure. Ascites, cirrhosis, hepatic coma, and hepatorenal syndrome may also exacerbate the clinical manifestations of acute HEV patients and HBV/HEV superinfected patients (Fan et al., 2021). Recently, some cases of persistent HEV infection and chronic active hepatitis associated with HEV have been found in patients undergoing solid organ transplantation, and chronic HEV infection can cause rapid and severe liver disease (Banas et al., 2006; Kamar et al., 2007; Gérolami et al., 2008; Kamar et al., 2008). Dalton et al. reported that chronic HEV infection might occur in HIV-infected patients and is associated with active hepatitis (Dalton et al., 2009). There are also cases of HEV superinfection with hepatitis A, hepatitis B, hepatitis C, *Campylobacter jejuni*, *Giardia intestinalis*, or malaria, which indicates that HEV infection may exacerbate liver damage, accelerate disease progression, and increase mortality in patients with cirrhosis or chronic liver disease (Lorenzo et al., 2016; Sahra et al., 2021; Butt et al., 2019; Tseng et al., 2020; Zitelli et al., 2021).

In this case, the patient was co-infected with HEV, *Clonorchis sinensis*, and bacterial infection, which has not been previously reported to the best of our knowledge. The pathogenesis of co-infection is still unclear and may be related to immune impairment. From this case and related studies, HEV should be considered in patients with jaundice as the main manifestation encountered in clinical practice and common diseases. In the diagnosis of acute hepatitis E patients, the detection of both HEV-IgM antibody and HEV RNA is necessary, since using any one of these markers alone may lead to misdiagnosis (Rivero-Juarez et al., 2021). Some new biomarkers, such as the HEV-Ag ELISA, are also easily implemented and may be especially useful in hyperendemic regions (Lytton et al., 2021).

Supportive therapy is still the mainstay treatment for hepatitis E since specific antiviral drugs are not available. Ribavirin may be beneficial to clear HEV-RNA, shorten the recovery time of liver function, and prevent the progression to liver failure. However, there are no clear treatment recommendations, and it may be more suitable for chronic HEV infection. If HEV replication persists for more than 3 months, ribavirin therapy should be considered, as well as IFNα therapy for patients who do not respond to ribavirin (Elisa et al., 2018; Kupke and Werner, 2021). Moreover, vaccines are no doubt the best protection against viral infections. Zhu and colleagues reported that a recombinant hepatitis E vaccine (HEV 239) is well tolerated and effective in preventing hepatitis E in the general population in China, but the vaccine is not available worldwide at this moment (Zhu et al., 2010). The Working Group did not recommend the routine use of this vaccine for pregnant women, patients with chronic liver disease, and immunocompromised persons because of uncertainties in safety and immunogenicity (World Health Organization, 2021; Marascio et al., 2022).

Furthermore, *Clonorchis sinensis* and *E.coli* are not rare; co-infection of both and HEV virus may threaten human health, especially in the elderly, pregnant women, immunocompromised patients, or those who have undergone post-solid organ transplantation and complicated with chronic diseases. Praziquantel is the first choice for the anthelmintic treatment of *Clonorchis sinensis*. In this case, after 2 weeks of treatment with albendazole tablets (because of a praziquantel shortage in the local hospital), parasites were repeatedly drained out and the bilirubin continued to rise; therefore, a course of praziquantel was prescribed as the first-line therapy. This indicates that further praziquantel therapy may be considered for *Clonorchis sinensis* infection if albendazole therapy fails. The patient showed reduced transaminases and increased bilirubin after the endoscopic nasobiliary drainage and albendazole therapy. It could be that lots of liver cells were necrotic and the ability to process bilirubin was progressively decreased, resulting in the increase of bilirubin. However, albendazole-induced liver injury might be a “possible” factor, according to the RUCAM/CIOMS (The Roussel Uclaf Causality Assessment Method of the Council of International Organization of Medical Science, RUCAM/CIOMS) score (4 points), while the elevated level of hepatic transaminases was one of the most common side effects of albendazole and the pattern of serum enzyme elevations was typically hepatocellular or mixed (Wilson, 2012).

Adult liver flukes can remain in the bile ducts for 26 years and may re-infect, so the cumulative parasite load in infected individuals may increase with age. Therefore, symptomatic infection is most common in middle-aged and elderly people and may be delayed for many years after the initial infection happened (Tan et al., 2009; Sripa et al., 2018). This patient had been exposed to untreated water for a long time, so the first-infection cannot be determined; nevertheless, a chronic infection should also be considered.

This case demonstrates the importance of monitoring HEV antibodies and HEV-RNA in the population of patients with abnormal liver function, especially those who are immunocompromised or have post-solid organ transplantation and chronic liver disease for co-infection with other pathogens. This report has some limitations. Due to limited detection, HEV genotype and HEV RNA quantitative tests were not completed. The changes in HEV RNA and HEV IgM should also be evaluated in different stages. In the future, more work on epidemiological tracing, HEV-RNA quantitative analysis, and HEV genotype detection, as well as the association between human and animal vaccinations, is needed.

## Data availability statement

The original contributions presented in the study are included in the article/supplementary material. Further inquiries can be directed to the corresponding author.

## Author contributions

All authors listed have made a substantial, direct, and intellectual contribution to the work and approved it for publication. LZ and XW contributed equally effort to this paper and share first authorship. ZW and DC are co-corresponding authors.
